# Serum C-Reactive Protein Is Negatively Associated With Olfactory Identification Ability in Older Adults

**DOI:** 10.1177/20416695211009928

**Published:** 2021-04-14

**Authors:** Ingrid Ekström, Davide Liborio Vetrano, Goran Papenberg, Erika J. Laukka

**Affiliations:** Aging Research Center, Department of Neurobiology, Care Sciences and Society, 27106 Karolinska Institutet and Stockholm University, Stockholm, Sweden; Aging Research Center, Department of Neurobiology, Care Sciences and Society, 27106 Karolinska Institutet and Stockholm University, Stockholm, Sweden; Centro di Medicina dell’Invecchiamento, IRCCS Fondazione Policlinico A. Gemelli, Rome, Italy; Aging Research Center, Department of Neurobiology, Care Sciences and Society, 27106 Karolinska Institutet and Stockholm University, Stockholm, Sweden; Aging Research Center, Department of Neurobiology, Care Sciences and Society, 27106 Karolinska Institutet and Stockholm University, Stockholm, Sweden; 225341 Stockholm Gerontology Research Center, Stockholm, Sweden

**Keywords:** inflammation, olfaction, odor, aging, CRP, sex differences

## Abstract

**Importance:**

Olfactory deficits are common in aging and associated with several conditions linked to inflammation. A few studies suggest that increased concentration of pro-inflammatory biomarkers may be related to olfactory deficits, but these associations are understudied in population-based samples.

**Objective:**

To investigate the association between serum concentrations of C-reactive protein (CRP) and olfactory identification level as well as rate of change in aging.

**Methods:**

We included 1,721 participants (mean age 70.5 years; 61.9% female) with at least two olfactory assessments across the 12-year follow-up. Baseline level and change in odor identification were estimated with linear mixed models as a function of CRP levels, derived from blood plasma at baseline.

**Results:**

Results indicated a negative dose–response association between CRP level and odor identification scores at baseline, after adjustment for demographic, cognitive, health, and lifestyle factors. CRP levels ranging between 11 and 20 mg/L were significantly related to lower olfactory ability (β = −0.811, 95% confidence interval [CI] [−1.503 to −0.118]; *p* = .022). Likewise, CRP values above 20 mg/L were related to lower olfactory scores, an association that approached statistical significance (β = −0.996, 95% CI [−2.045 to 0.054]; *p* = .063). We found no associations between CRP and olfactory change (*p*s > .368). Sensitivity analyses showed that associations between CRP and olfaction were confined to younger participants (age ≤72 years) and men (*p*s < .034).

**Conclusions:**

Our findings suggest a negative association between serum CRP levels and olfactory identification ability in aging that may be dependent on age and sex.

## Introduction

Although some olfactory loss is expected along with the aging process (Attems et al., 2015; Doty & Kamath, 2014), olfactory impairment is overrepresented in older adults with depressive symptoms (Kohli et al., 2016), cardiovascular disease (Seubert et al., 2017), diabetes mellitus (Lietzau et al., 2018), and neurodegenerative conditions, such as Alzheimer’s disease (AD) and Parkinson’s disease (Mesholam et al., 1998; Sun et al., 2012). Furthermore, olfactory impairment is associated with shorter survival among older adults (Devanand et al., 2015; Ekström et al., 2017; Pinto et al., 2014). This suggests that the olfactory system is vulnerable to processes associated with age-related conditions. One such process is inflammation. Indeed, a recent study suggests that the association of olfactory dysfunction and mortality in older adults may be mediated by inflammatory processes (Laudisio et al., 2019). Two studies that examined the link between inflammatory markers and olfactory function found elevated levels of cytokine interleukin-6 (IL-6) in blood plasma of participants with olfactory deficits (Cazzolla et al., 2020; Henkin et al., 2013). Furthermore, in patients with advanced renal impairment, olfactory deficits have been linked to higher levels of the inflammatory marker C-reactive protein (CRP; Raff et al., 2008). CRP is a liver-derived pentraxin and a highly sensitive serum biomarker of both acute and chronic inflammation as well as of tissue damage resulting from excessive inflammation or failure in the initial inflammatory response (Sproston & Ashworth, 2018). Elevated CRP levels are amongst others related to cardiovascular disease (CVD; Emerging Risk Factors Collaboration, 2012), stroke (Kuo et al., 2005; Luo et al., 2012), depression (Kuo et al., 2005; Valkanova et al., 2013), cognitive dysfunction (Kuo et al., 2005; Tegeler et al., 2016), and AD (Ng et al., 2018; Swardfager et al., 2010; Wehling et al., 2015), conditions in which olfactory impairment is also overrepresented (Croy et al., 2014; Kotecha et al., 2018; Roberts et al., 2016; Zou et al., 2016). However, to our knowledge, no study has so far examined whether CRP concentrations are associated with olfactory performance in a population-based sample of older adults. The aim of our study was to investigate the relationship between CRP levels and odor identification, both regarding olfactory ability at baseline and rate of olfactory change across 12 years of follow-up. Given that both inflammatory processes and olfactory function differ by age and sex (Doty & Kamath, 2014; Franceschi & Campisi, 2014; Roved et al., 2017; Sorokowski et al., 2019), sensitivity analyses tested associations between CRP and olfactory function in younger and older participants and females and males.

## Methods

### Participants

Participants were from the Swedish National Study on Aging and Care in Kungsholmen (SNAC-K), an ongoing longitudinal population-based study on aging and health that started in 2001 (Santoni et al., 2017). All parts of the SNAC-K project have been approved by the Ethics Committee at Karolinska Institutet or by the Regional Ethical Review Board in Stockholm, Sweden. The participants provided written informed consent. Of 4,590 invited participants, 3,363 participants enrolled in the baseline assessment. Of these, 2,848 completed a neuropsychological test battery and 2,569 underwent olfactory assessment (Larsson et al., 2016). We excluded participants with a diagnosis of dementia, schizophrenia, Parkinson’s disease, or developmental disorder at baseline, as well as those with a Mini-Mental State Examination (MMSE) score below 24, indicative of cognitive impairment. We further excluded 695 individuals who did not participate in at least one of the additional four olfactory assessments after baseline—age = 75.1 (10.6); 59.1% female; years of education = 11.2 (4.1); baseline odor identification score = 10.6 (3.6); and CRP = 6.1 (4.8). Comparisons between the final sample and participants without follow-up showed that the latter group was significantly older (*t* = −9.96, *p* < .001, Hedges’s *g* = −0.47), had fewer years of education (*t* = −5.4, *p* < .001, Hedges’s *g* = −0.25), had lower olfactory scores (*t* = −9.8, *p* < .001, Hedges’s *g* = −0.49), and had higher average CRP (*t* = 2.4, *p* = .017, Hedges’s *g* = 0.13). We found no difference in sex distribution between these groups (χ^2^ = 1.65, *p* = .199). Of the remaining 1,780 participants, 59 had not participated in the blood sampling assessment and information about CRP could not be obtained. The final sample consisted of 1,721 participants who participated in the study during an average follow-up time of 9.2 (3.1) years.

### Odor Identification Assessment

Odor identification was measured with the Sniffin’ Sticks, a well-established and norm-referenced olfactory test kit with high test–retest reliability (Hummel et al., 1997, 2007; Croy et al., 2015). The testing procedure has been described in detail elsewhere (Ekström et al., 2020). In brief, participants were presented with 16 household odors and instructed to freely identify the presented odor by providing a verbal descriptor (free identification). If they failed to provide a correct label, they were presented with four written response alternatives to choose the label that best matched the odor (cued identification). The odor identification score was calculated as the number of correctly identified odors with either free or cued identification, with a performance range of 0 to 16. Participants were randomized to three different presentation orders of the odor identification items at baseline and presentation order was rotated across follow-up occasions. Participants with obvious symptoms of viral infections or other respiratory problems did not perform olfactory testing.

### C-Reactive Protein

Non-fasting venous blood samples were obtained at the beginning of each individual assessment, centrifuged for 10 minutes at 3,000 runs per minute, and kept at 2°C. CRP serum concentrations were analyzed at Karolinska University Hospital, Stockholm, within 3 hours from sampling, through an enzyme-linked immunosorbent assays with a lower detection limit of 5 mg/L (i.e., low-sensitivity test). CRP was further categorized into the following four levels (Kravitz et al., 2009; Pepys & Hirschfield, 2003): (a) CRP of 0 to 5 mg/L, representing normal concentrations; used as the reference group; (b) CRP of 6 to 10 mg/L, representing somewhat elevated values that may, however, still be encountered in the healthy population; (c) CRP of 11 to 20 mg/L, representing clearly elevated concentrations that may be indicative of either acute or chronic inflammation; and (d) CRP of >20 mg/L, indicative of either acute or chronic inflammation.

### Assessment of Covariates

Age was measured as years since birth and education as years of formal schooling. These variables were centered on their respective means. We included a Swedish vocabulary test, SRB1, from the Synonym Reasoning Blocks (Dureman , 1960), with a similar format to the odor test, to control for semantic word knowledge.

Several diseases, health conditions, and behavioral/lifestyle factors that may be linked to inflammation were included as covariates. Diagnosis of diseases were made by physicians according to the International Classification of Diseases 10th edition (ICD-10) based on clinical examinations, medication lists, participant self-report, medical records, and information gathered from participants’ proxies. The diagnostic procedure is detailed elsewhere (Calderón-Larrañaga et al., 2017). We considered history of cerebrovascular disease, cardiovascular diseases including atrial fibrillation, heart failure, and ischemic heart disease, inflammatory bowel disease, inflammatory arthropathies, autoimmune disease, chronic kidney disease, and current depression. A diagnosis of diabetes Type 1 or 2 was obtained from medical history, use of diabetes drugs (ATC code A10), diagnosis in the inpatient register, or HbA1c ≥6.5% (48 mmol/mol) (Marseglia et al., 2019). We further included hypertension Stage 2 (≥160/100 mmHg or current use of antihypertensive medication), intake of corticosteroids (yes or no; ATC codes H02 and M01BA), smoking (current vs. previously or never), and longest held occupation (manufacturing vs. nonmanufacturing profession) as covariates.

### Statistical Analysis

We used linear mixed models with unstructured variance–covariance matrices to investigate olfactory level and change as a function of CRP levels at baseline. Time in study measured in years from baseline was used as the time scale. The random effects included random intercept and slope, allowing for individual differences at baseline and across time. Age, sex, education, and odor test version were adjusted in the basic model. We entered each of the additional covariates individually to the basic model to test if these were associated with olfactory ability. All covariates that were related to odor identification, based on an alpha-level < .10, were then added to the basic model to constitute the multiadjusted model. An alpha-level of <.05 was used in the main analyses. In sensitivity analyses, we excluded participants with CRP concentrations above 50 mg/L, indicative of acute infection. In further analyses, we stratified the sample according to age (≤72 vs. ≥78 years) and sex (female vs. male).

## Results

Background characteristics for the total sample as well as for age, sex, and CRP subgroups are summarized in Table 1. Results from the basic model (adjusted for demographics and odor test version) indicated a negative dose–response association between CRP levels and olfactory scores at baseline. Somewhat elevated CRP values, ranging between 5 and 10 mg/L, were not associated with olfactory function (*p* = .608). By contrast, participants with CRP concentrations between 11 and 20 mg/L had lower olfactory identification scores than the reference group, an association that was statistically significant (*p* = .020). Likewise, we observed lower olfactory scores for CRP concentrations above 20 mg/L, an association that approached statistical significance (*p* = .086). The lack of statistical significance was likely attributable to the small sample size with resulting low power in this group. Although olfactory function decreased significantly as a function of time (annual change rate of −0.213; *p* < .001), as described in detail elsewhere (Ekström et al., 2020), no statistically significant associations between CRP levels at baseline and olfactory change were observed (*p*s > .406).

**Table 1.** Participants Characteristics.

	Total sample *N* = 1,721	Age ≤ 72 *n* = 1,144	Age ≥ 78 *n* = 577	Female *n* = 1,063	Male *n* = 658	CRP < 5 mg/L *n* = 1,422	CRP 6–10 mg/L *n* = 222	CRP 11–20 mg/L *n* = 54	CRP > 20 mg/L *n* = 23
Time in study (years)	9.2 (3.1)	10.2 (2.4)	7.2 (3.2)	9.2 (3.0)	9.2 (3.1)	9.3 (3.0)	8.9 (3.1)	7.9 (3.2)	8.3 (3.4)
Number of follow-up assessments	2.0 (0.8)	1.9 (0.6)	2.2 (1.1)	1.9 (0.8)	2.0 (0.8)	2.0 (0.8)	1.9 (0.9)	1.7 (0.7)	2.1 (1.0)
Odor identification score, mean (*SD*)	12.1 (2.8)	12.7 (2.3)	10.7 (3.1)	12.3 (2.7)	11.7 (2.9)	12.1 (2.7)	12.0 (2.9)	10.9 (3.5)	10.5 (2.9)
Age, mean (*SD*)	71.0 (9.4)	65.2 (4.8)	82.4 (4.6)	71.8 (9.7)	69.7 (8.7)	70.6 (9.3)	72.5 (9.6)	72.9 (9.6)	74.0 (9.9)
Female, %	61.9	58.7	67.9	—	—	61.5	64.9	59.3	56.5
Years of education, mean (*SD*)	12.6 (4.2)	13.5 (4.2)	10.8 (3.8)	12.3 (4.2)	13.1 (4.3)	12.8 (4.3)	12.1 (4.3)	10.7 (2.9)	11.0 (3.5)
Manufacturing profession, %	17.6	12.6	27.5	20.7	12.5	17.2	20.3	14.8	21.7
Smoking, % current/ever/never	13.1/41.1/45.9	15.4/43.7/40.9	8.5/35.9/55.6	13.7/35.5/50.8	12.1/50.1/37.9	13.2/40.7/46.1	13.2/43.8/42.9	9.3/42.6/48.2	13.0/34.8/52.2
Vocabulary (SRB1), mean (*SD*)	23.5 (4.6)	24.3 (4.1)	21.8 (5.0)	23.5 (4.6)	23.5 (4.5)	23.6 (4.5)	22.9 (4.7)	22.6 (5.6)	23.2 (3.5)
History of cerebrovascular disease, %	7.1	3.9	13.3	7.1	7.1	6.8	6.8	14.8	8.7
Atrial fibrillation, %	6.3	3.1	12.8	5.4	7.9	5.9	6.8	13.0	13.0
Heart failure, %									
Coronary heart disease, %									
Diabetes, %	7.7	7.0	9.0	5.8	10.6	6.6	10.8	22.2	8.7
Hypertension, %	69.5	64.2	80.1	69.6	69.3	68.1	76.1	75.9	78.3
Inflammatory bowel disease, %	1.1	1.2	0.9	1.1	1.1	1.1	0.5	3.7	0.0
Autoimmune disease, %	4.5	3.4	6.6	4.9	3.8	3.9	7.2	7.4	8.7
Inflammatory arthropathies, %	3.5	3.0	4.5	3.3	3.8	2.7	6.8	5.6	13.0
Kidney disease, %	28.5	13.5	58.2	35.6	64.4	26.7	35.1	35.2	56.5
Depression, %	3.0	2.0	5.0	3.6	2.1	3.0	3.2	3.7	4.4
Corticosteroids use, %	3.0	2.0	4.9	3.3	2.4	2.1	7.2	1.9	17.4

*Note*. Percentage missing values: 0.5% for vocabulary and smoking. *SD* = standard deviation; *N* = sample size.

In addition to the covariates in the basic model, vocabulary, atrial fibrillation, hypertension, current smoking, depression, and intake of corticosteroids were significantly associated with olfactory scores in our sample (*p*s < .071) and were therefore included in the multiadjusted model. The pattern of results for the multi-adjusted model resembled closely that of the basic model. In comparison to the reference group, CRP levels of between 11 and 20 mg/L (*p* = .022), as well as of above 20 mg/L (*p* = .063), were associated with lower olfactory scores. Again, we found that associations in the latter group only approached significance, which is likely due to lack of power. As before, we observed no statistically significant associations between CRP levels and olfactory change trajectories (*p*s > .368). Coefficients and confidence intervals from the basic and the multiadjusted models are reported in Table 2.

**Table 2.** Results of Basic and Multiadjusted Linear Mixed Models Estimating Odor Identification Scores Based on CRP-Levels.

	Basic model ^a^		Multiadjusted model ^b^	
	Cross-sectional β (95% CI); *p*-value	Predictor × Time β (95% CI); *p*-value	Cross-sectional β (95% CI); *p*-value	Predictor × Time β (95% CI); *p*-value
Total sample (*N* = 1,721)				
CRP: 5–10 mg/L; *n* = 222	0.095 (−0.267 to 0.456); .608	−0.023 (−0.076 to 0.031); .406	0.084 (−0.278 to 0.447); .649	−0.010 (−0.064 to 0.044); .724
CRP: 11–20 mg/L; *n* = 54	−0.832 (−1.533 to −0.131); .020	0.044 (−0.068 to 0.157); .439	−0.811 (−1.503 to −0.118); .022	0.040 (−0.072 to 0.151); .487
CRP: >20 mg/L; *n* = 23	−0.910 (−1.947 to 0.127); .086	−0.056 (−0.219 to 0.106); .496	−0.996 (−2.045 to 0.054); .063	−0.075 (−0.239 to 0.088); .368
Participants with CRP < 50 mg/L (*n* = 1,716)				
CRP: 5–10 mg/L; *n* = 222	0.095 (−0.267 to 0.457); .606	−0.023 (−0.076 to 0.031); .407	0.087 (−0.275 to 0.450); .528	−0.010 (−0.064 to 0.044); .724
CRP: 11–20 mg/L; *n* = 54	−0.832 (−1.533 to −0.131); .020	0.044 (−0.068 to 0.157); .439	−0.811 (−1.504 to −0.119); .022	0.040 (−0.072 to 0.151); .487
CRP: >20 mg/L; *n* = 18	−0.969 (−2.138 to 0.200); .104	−0.103 (−0.286 to 0.081); .272	−1.045 (−2.203 to 0.113); .077	−0.104 (−0.287 to 0.079); .264

CRP = C-reactive protein; CI = confidence interval.

^a^Adjusted for age, sex, years of education, and test version of odor identification task.

^b^Adjusted for age, sex, years of education, test version of odor identification task, vocabulary (SRB1), atrial fibrillation, hypertension, depression, smoking, and intake of corticosteroids.

### Sensitivity Analyses

Excluding participants with CRP ≥ 50 mg/L (*n* = 5), likely indicative of acute infection, did not affect our results (Table 2). In stratified, multiadjusted analyses, we obtained significant associations between CRP values above 10 mg/L, as well as above 20 mg/L, and poorer odor identification for the younger participants (*p*s < .034). Stratified analyses also showed significant associations between CRP values above 10 mg/L and lower olfactory scores for male participants (*p*s < .014). By contrast, we found no significant associations neither for participants aged ≥ 78 nor for women (*p*s > .249). As in the total sample, no statistically significant interaction effects with time were found for the subsamples (*p*s > .294). The results of the stratified multiadjusted analyses are summarized in Table 3.

**Table 3.** Results of Multiadjusted Linear Mixed Models Estimating Odor Identification Scores Based on CRP-Levels in Subsamples.^a^

	Cross-sectional β (95% CI); *p*-value	Predictor × Time β (95% CI); *p*-value	Cross-sectional β (95% CI); *p*-value	Predictor × Time β (95% CI); *p*-value
	**Age ≤ 72 (*n* = 1,144)**		**Age ≥ 78 (*n* = 577)**	
CRP: 5–10 mg/L	−0.013 (−0.423 to 0.396); .949	−0.015 (−0.075 to 0.045); .630	−0.147 (−0.522 to 0.816); .666	0.044 (−0.064 to 0.152); .425
CRP: 11–20 mg/L	−1.091 (−1.841 to −0.341); .004	−0.005 (−0.120 to 0.110); .935	−0.201 (−1.604 to 1.202); .779	0.209 (−0.084 to 0.502); .162
CRP: >20 mg/L	−1.594 (−3.064 to −0.124); .034	−0.002 (−0.227 to 0.223); .984	−0.927 (−2.503 to 0.650); .249	−0.023 (−0.264 to 0.218); .851
	**Female (*n* = 1,063)**		**Male (*n* = 658)**	
CRP: 5–10 mg/L	0.142 (−0.293 to 0.576); .523	−0.020 (−0.088 to 0.047); .555	−0.008 (−0.655 to 0.640); .982	0.007 (−0.083 to 0.096); .883
CRP: 11–20 mg/L	−0.420 (−1.281 to 0.442); .340	0.023 (−0.115 to 0.162); .740	−1.448 (−2.609 to −0.287); m .014	0.063 (−0.127 to 0.254); .515
CRP: >20 mg/L	−0.539 (−1.911 to 0.833); .442	−0.039 (−0.245 to 0.167); .713	−0.151 (−3.171 to 0.151); .075	−0.144 (−0.413 to 0.125); .294

CRP = C-reactive protein; CI = confidence interval.

^a^Adjusted for age, sex, years of education, test version of odor identification task, vocabulary (SRB1), atrial fibrillation, hypertension, depression, smoking, and intake of corticosteroids.

## Discussion

In this population-based study of older adults, we observed a negative dose–response cross-sectional association between CRP levels and olfactory identification scores. The observed effects were independent from demographic, cognitive, health, and lifestyle factors associated with olfactory ability or inflammation. Likewise, results of sensitivity analysis showed that the effects were not driven by participants with CRP levels above 50 mg/L, indicative of an acute infection.

Our results indicate a gradual decrease of olfactory scores as a function of increasing CRP-levels. Although normal CRP levels for adults may be population-dependent and have not been precisely defined, values of 5 mg/L and above are typically considered elevated in clinical settings (Kravitz et al., 2009; Pepys & Hirschfield, 2003). However, concentrations between 5 and 10 mg/L may still be found among the healthy older population (Franceschi & Campisi, 2014). This may explain why participants in this group did not exhibit significantly lower olfactory scores in our sample. By contrast, statistically significant associations were found for CRP values of 11 to 20 mg/L. The association between CRP and olfaction was even more pronounced among participants with values above 20 mg/L. Given the comparatively small sample size, a lack of statistical significance may likely be due to low power in this group. Further studies with larger sample sizes are therefore needed to confirm this potential association.

CRP values within the observed range can be encountered among individuals with both chronic and acute inflammation (Kravitz et al., 2009; Pepys & Hirschfield, 2003; Ribeiro, 1997). Importantly, however, it should be stressed that participants were not acutely ill at the time of assessment. We suggest therefore that the observed associations may, at least in part, be representative of a relationship between chronically elevated CRP levels and olfaction. Studies mapping changes in both CRP and olfaction are needed to evaluate this further.

Our findings align with a previous study of 31 patients with advanced renal impairment that found significantly higher CRP levels in those with lower olfactory scores (Raff et al., 2008). Furthermore, previous studies found elevated levels of the cytokine IL-6 in blood plasma of participants with olfactory deficits (Henkin et al., 2013; Laudisio et al., 2019). IL-6 is a primary determinant of hepatic production of CRP and plays a key role in acute-phase inflammatory responses to for example viral or bacterial infections (Heinrich et al., 1990). Given its pro-inflammatory properties, it is also elevated in many chronic diseases characterized by a low-grade inflammation (Gabay, 2006; Maggio et al., 2006; Naugler & Karin, 2008). Interestingly, the association between CRP levels and olfactory function observed in the current study was independent from co-occurrent chronic conditions. To note, a low-grade inflammation—referred to as inflammaging—is a common finding in older adults also in the absence of major diseases (Franceschi & Campisi, 2014).

Contrasting our cross-sectional observations, we found no evidence for longitudinal associations between baseline CRP concentrations and rate of olfactory decline. These findings may result from small effect-sizes that are especially difficult to detect in longitudinal analyses. Alternatively, they may indicate an effect of inflammation on olfaction that is exerted earlier in life and that is not related to olfactory changes in old age. A lack of longitudinal studies on CRP and olfaction hinders comparison with previous research.

Given that CRP is part of a complex and sophisticated immune response system, the observed associations between inflammatory markers and olfactory function may be explained by multiple potential mechanisms. For example, inflammation may induce changes within the olfactory system through an increased activation of microglia. Microglia are supportive glial cells with an important immune surveillance role (Aloisi, 2001) that regulate CRP (Juma et al., 2011). As the olfactory system is directly exposed to the outside environment, xenobiotics (e.g., virus, bacteria, pollutants) enter the brain via the olfactory pathway where they sometimes induce microgliosis, changes in microglia with an increased secretion of cytokines and inflammatory mediators as a result (Seo et al., 2018). In a normal state, microglia contribute to neurogenesis (Tremblay et al., 2011), whereas microgliosis-derived pro-inflammatory processes reduce the number of newborn cells in neurogenic centers, such as the olfactory bulb (Lazarini et al., 2012; Pickering et al., 2005). Indeed, previous results showed that IL-6 levels related to hyposmia were higher in nasal mucus than in plasma, urine, or saliva, indicating that biochemical changes induced by local inflammatory processes may underlie parts of olfactory loss (Cazzolla et al., 2020). In addition to local inflammation, the olfactory system may also be vulnerable to effects of systemic inflammatory changes. These changes may be induced by, for example, neurodegenerative or cardiovascular diseases that are accompanied by an increase in microglial activation (Badoer, 2010; Kingwell, 2012; Perry et al., 2010). Importantly, inflammatory markers are a highly heterogenous group that, in complex interaction with each other, perform a variety of biological functions (Slaats et al., 2016). The investigation of single inflammatory markers is thus insufficient to draw conclusions regarding underlying mechanisms. Recent work suggests that one may discriminate between different types of inflammatory reactions based on marker profiles (Holub et al., 2013; van de Veerdonk et al., 2012). To further our understanding about the mechanisms underlying inflammation in olfactory deficits, future studies may investigate a wide array of both pro- and anti-inflammatory markers, as well as their interactions.

Interestingly, sensitivity analyses showed that associations between CRP and olfactory function were confined to participants aged 72 years and below and males. Increasing age is a principal cause for olfactory loss (Doty & Kamath, 2014; Ekström et al., 2020), which may over-shadow potential effects of inflammatory processes on olfactory ability in older age groups. Alternatively, and as mentioned earlier, potential influences of inflammation on olfaction may be exerted before a certain age.

Female sex has consistently been shown to be associated with better olfactory performance (Sorokowski et al., 2019), but mechanisms behind this effect are poorly understood. A tentative hypothesis is that female sex may to some extent be protective against inflammation-induced olfactory loss. Indeed, sex differences in the human immune response are well-documented (Klein & Flanagan, 2016; Roved et al., 2017). The prognosis for females suffering from many inflammatory conditions is generally better than for men throughout the lifespan, which may result from a more efficient inflammatory response for females than for males facing similar insults (Casimir & Duchateau, 2011). For example, female renal patients have a better outcome than males, suggesting a cardioprotective effect of sex hormones on inflammation in the face of vascular injury (Stenvinkel et al., 2002). Importantly, inflammatory reactions are largely driven by hormonal status with estrogen exerting an inhibitory activity on microglia (Vegeto et al., 2008).

Main strengths of this study include the large population-based sample and access to both cross-sectional and longitudinal olfactory data. Several limitations of our study deserve mention. First, the measured serum levels of CRP can neither differentiate between local and systemic inflammation nor between central nervous system- and peripheral inflammation. Second, single time-point assessments of CRP cannot differentiate between chronic or acute inflammation and may be less reliable than repeated measures. Third, selective drop-out resulting from the longitudinal study design likely introduced bias to our results. Participants with a comparatively better sense of smell and lower average CRP were more likely to remain in our sample, which may lead to an underestimation of the actual association between inflammation and olfactory function.

## Conclusion

We found higher CRP levels in blood plasma to be associated with lower olfactory identification ability in older adults. Our results suggest olfactory function to be associated with inflammation. Further research considering a wider array, as well as multiple repeated measures, of inflammatory biomarkers are needed to further our understanding about these associations.
